# A Super-Ecliptic, pHluorin-mKate2, Tandem Fluorescent Protein-Tagged Human LC3 for the Monitoring of Mammalian Autophagy

**DOI:** 10.1371/journal.pone.0110600

**Published:** 2014-10-23

**Authors:** Isei Tanida, Takashi Ueno, Yasuo Uchiyama

**Affiliations:** 1 Department of Biochemistry and Cell Biology, National Institute of Infectious Diseases, Shinjuku, Tokyo, Japan; 2 Laboratory of Proteomics and Biomolecular Science, Research Support Center, Juntendo University Graduate School of Medicine, Bunkyo, Tokyo, Japan; 3 Department of Cellular and Molecular Neuropathology, Juntendo University School of Medicine, Bunkyo, Tokyo, Japan; Iowa State University, United States of America

## Abstract

Tandem fluorescent protein-tagged LC3s that were comprised of a protein tag that emits green fluorescence (e.g., EGFP or mWasabi) fused with another tag that emits red fluorescence (e.g. mCherry or TagRFP) were used for monitoring the maturation step of mammalian autophagosomes. A critical point for this tandem fluorescent-tagged LC3 was the sensitivity of green fluorescence at an acidic pH. EGFP and mWasabi continue to emit a weak, but significant, fluorescence at a pH of approximately 6. To overcome this issue, we focused on super-ecliptic pHluorin, which is a more pH-sensitive GFP variation. The green fluorescence of EGFP and mWasabi in the cells was still observed at weakly acidic levels (pH 6.0–6.5). In contrast, the fluorescence of pHluorin was more significantly quenched at pH 6.5, and was almost completely abolished at pH 5.5–6.0, indicating that pHluorin is more suitable for use in a tandem fluorescent protein-tag for monitoring autophagy. A pHluorin-mKate2 tandem fluorescence protein showed pH-sensitive green fluorescence and pH-resistant far-red fluorescence. We therefore generated expression plasmids for pHluorin-mKate2-tagged human LC3 (PK-hLC3), which could be used as a modifier for LC3-lipidation. The green and far-red fluorescent puncta of PK-hLC3 were increased under starvation conditions. Puncta that were green-negative, but far-red positive, were increased when autolysosomes accumulated, but few puncta of the mutant PK-hLC3ΔG that lacked the carboxyl terminal Gly essential for autophagy were observed in the cells under the same conditions. These results indicated that the PK-hLC3 were more appropriate for the pH-sensitive monitoring of the maturation step of autophagosomes.

## Introduction

Autophagy is a bulk process involving degradation of the cytosol, which includes organelles [1], [2]. During autophagy, isolation membranes/preautophagosomes are formed and elongated to engulf the cytosolic components. After elongation of the membranes, the isolation membranes are enclosed to form autophagosomes. Lysosomes are fused with autophagosomes to form autolysosomes. At this step, the lumenal pH of an autophagosome is acidified by the fusion with lysosomes, since the lumenal pH of a lysosome is 4.0–5.0. Intra-autophagosomal contents in autolysosomes are degraded by lysosomal hydrolases.

LC3 (microtubule-associated protein 1A and 1B light-chain 3; MAP-LC3/MAP1-LC3/MAP1A1B-LC3) is a unique modifier for ubiquitylation-like conjugation to localize to autophagosomes; LC3 is synthesized as proLC3. The carboxyl terminus of proLC3 is cleaved by Atg4B, a cysteine protease, to expose its carboxyl terminal Gly, which is essential for its conjugation reaction [3], [4], [5]. During autophagy, the cytosolic LC3 (LC3-I) is activated by Atg7 (an E1-like enzyme), transferred to Atg3 (an E2-like enzyme), and finally conjugated to phospholipids (phosphatidylethanolamine and/or phosphatidylserine) to form the LC3-phospholipid conjugate (LC3-II) [6], [7], [8], [9]. LC3-II is localized to autophagosomes [4]. During the fusion of autophagosomes with lysosomes, LC3-II on the cytosolic face of autophagosomes is delipidated by Atg4B to form LC3-I, and LC3-II on the lumenal face of autophagosomes is degraded by lysosomal hydrolases. Therefore, LC3-II is a promising autophagosomal marker, and the lysosomal turnover of LC3-II is a marker for autophagic activity [4], [10].

The formation of puncta by fluorescent protein-tagged LC3s (FP-LC3s) (EGFP-LC3, YFP-LC3, CFP-LC3, RFP-LC3, mCherry-LC3 and HcRed-LC3) are used to monitor autophagosome formation [11], [12], [13], [14], [15], [16]. When autophagy is induced in the cells expressing a FP-LC3, the FP-LC3 is lipidated and localized to autophagosomes. The localization of FP-LC3 to autophagosomes was recognized as appropriate fluorescent puncta. Therefore, an increase in the puncta of FP-LC3 reflects an increase in autophagosomes and autolysosomes in the cells. The inhibition of lysosomal degradation results in a further increase in the puncta of FP-LC3, since autolysosomes in addition to autophagosomes are significantly accumulated by the inhibition [10]. An increase in the puncta that is promoted by the inhibition of lysosomal degradation is considered to be a reflection of autophagic flux. Mutant FP-LC3ΔGs (EGFP-LC3ΔG, YFP-LC3ΔG, CFP-LC3ΔG, and HcRed-LC3ΔG) lacking the carboxyl terminal Gly that is essential for LC3 lipidation are used as negative controls [12].

The FP-LC3s are considered to be a useful tool for the monitoring of autophagy, but there are limitations. It is difficult to use the fluorescent puncta of FP-LC3 to distinguish between autophagosomes and autolysosomes. In addition, Kimura *et al.* have found that EGFP-LC3 tends to lose fluorescence due to lysosomal acidic and degradative conditions, but mRFP-LC3 does not, indicating that the former mostly reflects only autophagosomes. The difference between EGFP-LC3 and mRFP-LC3 is a dependence on their pKa (pKa of EGFP is 5.9, and that of mRFP is 4.5) in addition to a degradation of EGFP by the lysosomal contents [17].

Based on these findings, a mRFP-EGFP tandem fluorescent protein-tagged LC3 (tfLC3) was generated for the monitoring of the autophagosomal maturation step [17]. The green and red double-positive fluorescent puncta of tfLC3 reflect autophagosomes (non-acidic compartments). The fluorescent puncta that are green-negative and red-positive reflect autolysosomes as acidic compartments, since EGFP tends to decrease its fluorescence at an acidic pH. An mCherry-EGFP-LC3 also was generated [18]. However, under acidic conditions (pH 4.0–5.0), EGFP has a weak fluorescence and acidic lysosomes [19], [20]. Therefore, because the green fluorescent puncta of EGFP in tfLC3 still partially reflects autolysosomes in addition to autophagosomes, the pH-sensitivity of green fluorescent protein is important in order to distinguish autophagosomes from autolysosomes with a higher degree of sensitivity when using tfLC3.

To improve the problem of an EGFP-based tandem fluorescent protein-tagged LC3, mTagRFP-mWasabi-LC3 was generated, since the pKa of mWasabi (pKa = 6.5) was higher than that of EGFP [21], [22]. mWasabi is a mutant of mTFP1, and mTFP1 is a pH-stable fluorescent protein [22], [23]. As yet, the mechanism by which the fluorescence of mWasabi decreases under acidic conditions remains unknown, while the pKa of mWasabi has been reported.

The higher pH-sensitivity of a protein tag, which emits green fluorescence, is a critical point for monitoring the autophagosomal maturation step using a tandem, fluorescent, protein-tagged LC3. Therefore, we focused on a pH-sensitive green fluorescent protein, super-ecliptic pHluorin (pKa = 7.6) (hereafter simply referred to as pHluorin) [24]. In the present study, we compared the pH-sensitivity of green fluorescent proteins, including EGFP, mWasabi, and pHluorin, and constructed a tandem, fluorescent, protein-tagged LC3 and its negative control mutant using the most pH-sensitive protein.

## Results

### The pH-sensitive green fluorescent protein, super-ecliptic pHluorin, is the most sensitive to acidic pH among EGFP, mWasabi, and pHluorin

**Figure 1:**
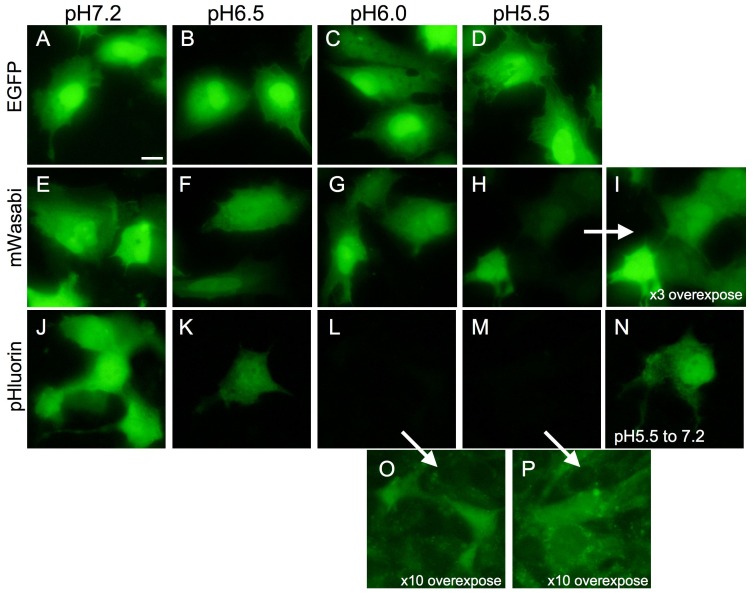
The pH sensitivity of the green fluorescence of EGFP, mWasabi, and super-ecliptic pHluorin. The green fluorescent proteins, EGFP (A–D), mWasabi (E–I), and pHluorin (J–P), were expressed in the Huh7.5.1 cells. After fixation and permeabilization of the cells, the cells were buffered at pH 5.5 (D, H, I, M, N, P), 6.0 (C, G, L, O), 6.5 (B, F, K), and 7.2 (A, E, J). The green fluorescence of each of the proteins in the cells were obtained with Biozero BZ-8000 using the filter set for GFP. The Images in A–H and J–N were obtained under the same conditions of the fluorescent microscope. The image in I was exposed three times longer than that in H. The images in O and P were exposed ten times longer than those in the respective L and M. In H, cells were incubated at pH 5.5, and the pH was changed to 7.2. Bar indicates 10 µm.

To investigate which is the most sensitive to acidic pH among EGFP, mWasabi, and pHluorin, we expressed each protein in Huh7.5.1 cells. After fixation, cells were permeabilized by digitonin, and the fluorescence derived from the fluorescent proteins was investigated with buffering at pH 5.5, 6.0, and 6.5 (Fig. 1). The fluorescence of EGFP was observed at pH 5.5–6.5 in addition to pH 7.2 (Fig. 1A–D). The fluorescence of mWasabi was slightly weakened at pH 6.0 and 6.5, and significantly decreased at pH 5.5 (Fig. 1E–H). The decreased fluorescence of mWasabi at pH 5.5 was detected via a three-time overexposure (Fig. 1H vs. I). The fluorescence of pHluorin was decreased significantly at pH 6.5, and little fluorescence was observed at pH 5.5 and 6.0 (Fig. 1L & M). A faint residual fluorescence of pHluorin was recognized at pH 5.5 and 6.0 using a ten-fold overexposure, while an autofluorescence of the cells was also observed under these conditions (Fig. 1O & P). When the pH of the incubated buffer was changed from 5.5 to 7.0, the green fluorescence of pHluorin was recovered (Fig. 1N). These results indicated that pHluorin was the most sensitive to an acidic pH among the three green fluorescent proteins.

### pHluorin-mKate2 fusion protein showed pH-sensitive green fluorescence and pH-resistant far-red fluorescence

**Figure 2:**
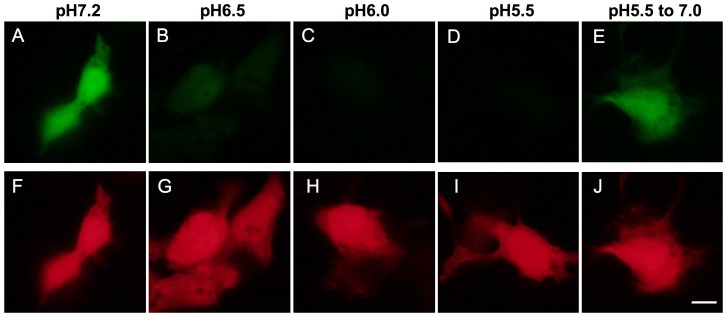
The pH sensitivity of the pHluorin-mKate2 tandem fluorescent protein. The pHluorin-mKate2 tandem fluorescent protein was expressed in the Huh7.5.1 cells. After fixation and permeabilization, cells were buffered at pH 5.5 (D, I), 6.0 (C, H), 6.5 (B, G), and 7.2 (A, F). In E and J, cells were incubated at pH 5.5, and the pH was changed to pH 7.2. The green and far-red fluorescence in the cells were obtained with Biozero BZ-8000 using the filter sets for GFP (A–E) and Texas Red (F–J). Bar indicates 10 µm.

Using pHluorin, we next generated a plasmid for the expression of pHluorin-mKate2 (green and far-red) tandem fluorescent protein in order to investigate whether the pHluorin-mKate2 fusion protein would show both pH-sensitive green fluorescence derived from pHluorin and pH-stable far-red fluorescence derived from mKate2. A pHluorin-mKate2 protein was expressed in Huh7.5.1 cells. After fixation and mild permeabilization, cells were buffered at pH 5.5–6.5, and the green and far-red fluorescence of pHluorin-mKate2 in the cells was monitored (Fig. 2). As expected, both green and far-red fluorescence was observed at pH 7.2 (Fig. 2A & F). The green fluorescence of pHluorin-mKate2 was significantly decreased at pH 6.5 and almost completely abolished at pH 5.5, while its far-red fluorescence was easily detected even at pH 5.5 (Fig. 2B–D vs. G–I). The green fluorescence was recovered when cells were incubated at pH 7.2 after incubation at pH 5.5 (Fig. 2E). These results indicated that the tandem fluorescent protein, pHluorin-mKate2, showed pH-sensitive green and pH-resistant far-red fluorescence.

### pHluorin-mKate2-tagged hLC3 is a modifier for LC3-conjugation

**Figure 3:**
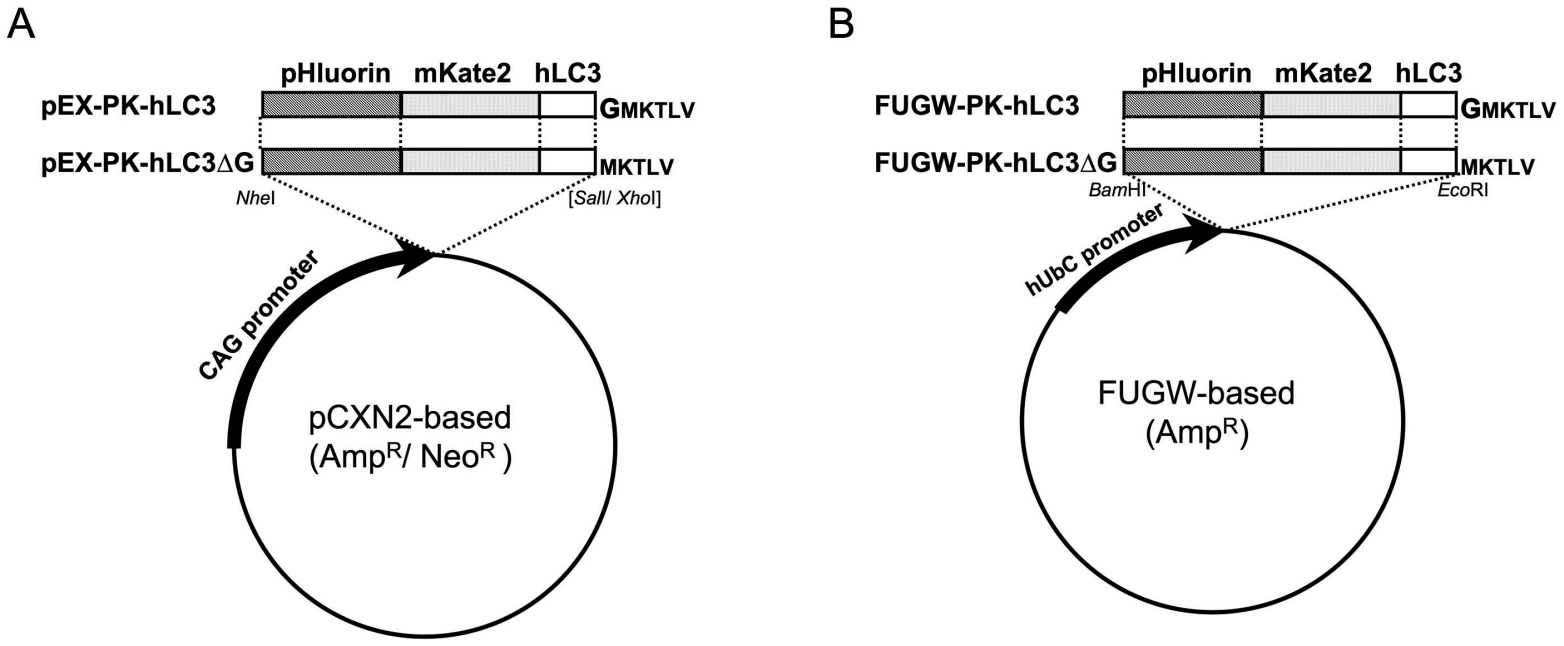
Schematic representation of the expression plasmids for wild type LC3 and mutant LC3ΔG fused to fluorescent proteins at the N-terminus. (A) The expression plasmids for wild type PK-hLC3, and mutant PK-hLC3ΔG under the control of chicken ß-actin (CAG) promoter. The name designated to each plasmid is shown in the left panel. (B) The plasmids for lentiviral packaging and transient expression for wild type PK-hLC3 and mutant PK-hLC3ΔG under the control of human polyubiquitin C (hUbC) promoter.

We then constructed mammalian expression plasmids for pHluorin-mKate2-tagged human LC3, which was designated PK-hLC3, under the control of a CAG promoter (Fig. 3A). As a negative control, we generated mammalian expression plasmids for PK-hLC3ΔG, which lacked the carboxyl terminal Gly of PK-hLC3 that is essential for LC3-lipidation. A FUGW plasmid is an expression vector for a 3^rd^ generation lentiviral expression system [25]. A set of FUGW-based plasmids for the expression of PK-hLC3 and PK-hLC3ΔG under the control of the human polyubiquitin promoter C were also generated (Fig. 3B).

**Figure 4:**
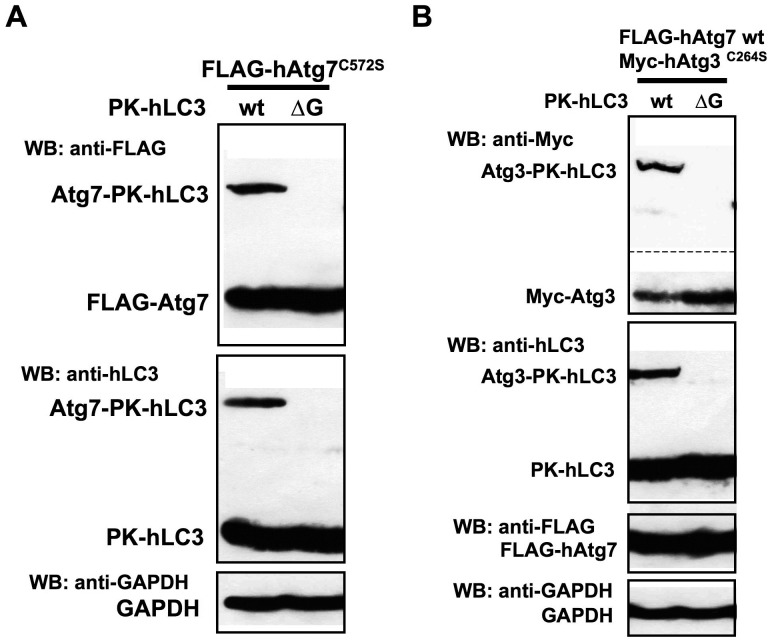
The formation of the Atg7-LC3 E1-substrate and Atg3-LC3 E2-substrate intermediates of fluorescent protein-tagged LC3. **(A)** The formation of the E1-substrate intermediate of Atg7 with fluorescent protein-tagged LC3s. The PK-hLC3** (wt) **was expressed together with FLAG-tagged human **Atg7** ^C572S^ in the Huh7.5.1 cells. After preparation of the cell lysate, total proteins were separated on SDS-PAGE. FLAG-hAtg7^C572S^ and PK-hLC3 were recognized by immunoblotting with appropriate antibodies. As a negative control, mutant PK-hLC3ΔG (Δ**G**) was expressed. As a loading control,** GAPDH** was employed. **Atg7-PK-hLC3** indicated the Atg7-LC3 (E1-substrate) intermediate with **Atg7** ^C572S^. **(B)** The formation of the E2-substrate intermediate of Atg3 with fluorescent protein-tagged LC3s. The PK-hLC3 was expressed together with wild type FLAG-tagged Atg7 and mutant Myc-Tagged Atg3^C264S^ in the Huh7.5.1 cells. FLAG-hAtg7, Myc-Atg3^C264S^ and PK-hLC3 were recognized by immunoblotting with appropriate antibodies. As a negative control, mutant PK-hLC3ΔG was expressed. As a loading control, **GAPDH** was employed. **Atg3-PK-hLC3** indicated their Atg7-LC3 intermediates with **Atg3** ^C264S^.

We investigated whether PK-hLC3 can form the Atg7-LC3 (E1-substrate) intermediate with Atg7 (Fig. 4A). When LC3-I activates Atg7, a transient E1-substrate intermediate is formed *via* a thioester bond between the carboxyl terminal Gly of LC3-I and the active site Cys^572^ in human Atg7 [7], [26]. The E1-substrate intermediate is unstable in the cells, since LC3-I is transferred to Atg3. Therefore, an active site mutant Atg7^C572S^ was employed to detect the formation of the E1-substrate intermediate. This mutant formed a stable E1-substrate intermediate *via* an *O*-ester bond with LC3 via Ser^572^ within the mutant Atg7^C572S^ and the carboxyl Gly within LC3 [7]. The Atg7^C572S^-LC3 intermediate was recognized by immunoblotting using appropriated antibodies when wild type PK-hLC3 was expressed together with Atg7^C572S^ (Fig. 4A, wt). In contrast, no intermediate was recognized when PK-hLC3ΔG was expressed instead of the wild type (Fig. 4A, wt vs. ΔG).

We next investigated the formation of the E2-substrate intermediate of PK-hLC3 with Atg3 (Fig. 4B). The Atg3-LC3 (E2-substrate) intermediate is unstable, and LC3 is conjugated to phospholipids. To investigate if PK-hLC3 can form an E2-substrate intermediate with Atg3, we employed an active-site mutant, Atg3^C264S^, since the mutant Atg3 forms an E2-substrate intermediate with LC3 *via* an *O*-ester bond instead of a thioester bond [8]. The Atg3-LC3 E2-substrate intermediate was recognized when wild type PK-hLC3 was expressed together with Atg3^C264S^ and Atg7. A scant level of E2-substrate intermediate was recognized when mutant PK-hLC3ΔG was expressed instead of wild type. These results demonstrated that PK-hLC3 has the ability to modify LC3-conjugation, and that mutant PK-hLC3ΔG is a suitable negative control of PK-hLC3.

### Puncta of PK-hLC3 were increased during autophagy

**Figure 5:**
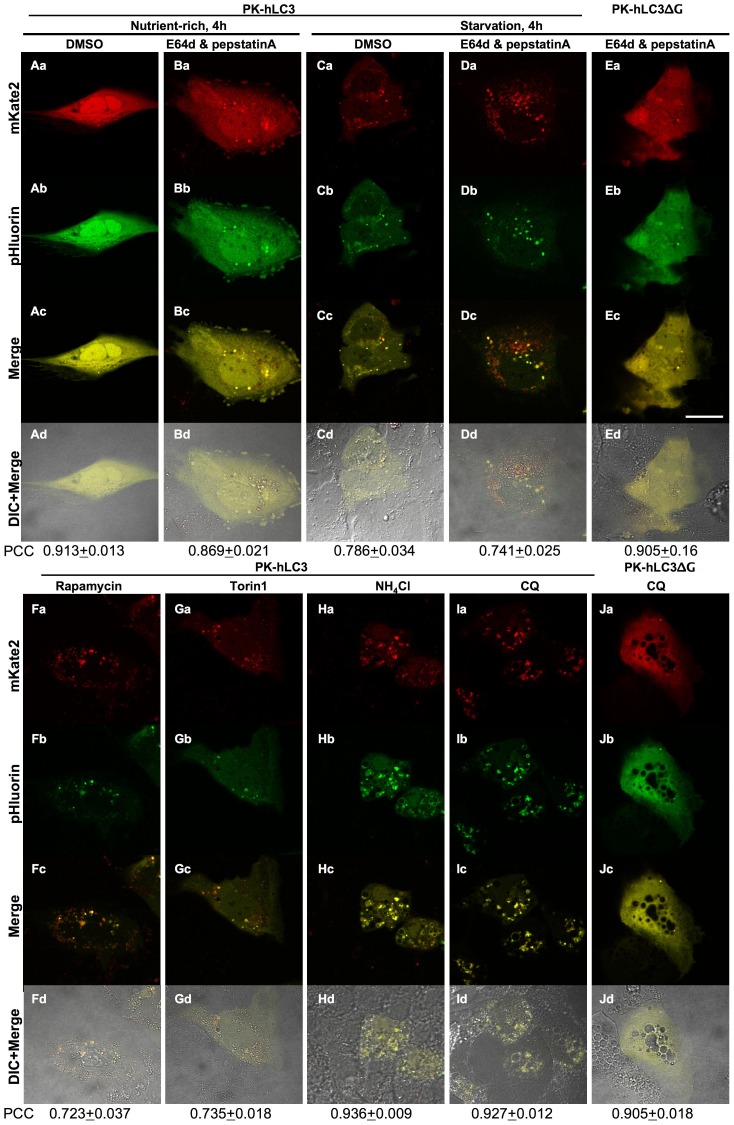
Formation of the puncta of PK-hLC3 during autophagy. The PK-hLC3 was expressed in Huh7.5.1 cells. The cells were incubated in the Krebs-Ringer buffer for 4 h as starvation conditions in the presence (**E64d & pepstatin A**) (Da–Dd) or absence (**DMSO**) (Ca–Cd) of 10 µg/ml E64d and 10 µg/ml pepstatin A (**Starvation, 4 h**). As nutrient-rich conditions, cells were incubated in the cultured medium (**Nutrient-rich, 4 h**) (Aa–Ad, Ba–Bd). For induction autophagy by the inhibition of mTOR-signaling pathway, cells were incubated in the cultured medium for 6 h in the presence of 100 nM **rapamycin** (Fa–Fd) or 100 nM **torin1** (Ga–Gd). To inhibit the fusion of autophagosome with lysosome, 20 mM ammonium chloride (**NH_4_Cl**) (Ha–Hd) and 20 µg/ml chloroquine (**CQ**) (Ia–Id) were treated to the cells incubated in the Krebs-Ringer buffer for 4 h. The **PK-hLC3ΔG** (Ea–Ed and Ja–Jd) was expressed in the cells instead of the PK-hLC3 under the same conditions (Da–Dd and Ia–Id, respectively). The far-red (**mKate2)** and green (**pHluorin)** fluorescence in the cells were monitored using a Olympus FluoView FV1000 confocal laser scanning microscope. “**Merge**” indicates the merging of the green (**pHluorin)** and far-red images (**mKate2**), and “**DIC+Merge**” indicates the overlaying the merged images on the DIC (differential interference contrast) images in the same field. Pearson's correlation coefficient (**PCC**) analysis with Costes' method was used as a measure of colocalization of mKate2 signals with pHluorin signals. The mean PCC value ± S.E. of at least 20 cells is shown on the bottom.

To investigate whether the PK-hLC3 can form puncta under starvation conditions, we next examined the formation of PK-hLC3 puncta under starvation conditions (Fig. 5). If PK-hLC3 was lipidated and localized to autophagosomes like endogenous LC3, the green and far-red fluorescent puncta of PK-hLC3 could be detected in the cells expressing PK-hLC3 during autophagy. Huh7.5.1 cells expressing PK-hLC3 were incubated in a Krebs-Ringer buffer for 4h to simulate starvation conditions that would induce autophagy. PK-hLC3 puncta of green and far-red fluorescence was investigated. Under nutrient-rich conditions, a few green and far-red, double-positive, fluorescent puncta were observed in the cells (Fig. 5Aa–Ad). Under starvation conditions, the double-positive puncta were increased (Fig. 5Ca–Cd). The green-negative, but far-red-fluorescent-positive, puncta were also increased in the cells (Fig. 5Ca–Cd), indicating that PK-hLC3 forms intracellular puncta under starvation conditions.

To further investigate whether the green-negative, but far-red-positive, puncta of PK-hLC3 increase when autolysosomes are increased, we examined the formation of puncta in the cells expressing PK-hLC3 in the presence of inhibitors for major lysosomal proteases and cathepsins under starvation conditions. E64d is an inhibitor for cathepsins B, H and L, and pepstatin A is an inhibitor for cathepsin D [27], [28], [29]. Inhibition of the protease activities of these cathepsins leads to an inactivation of lysosomal hydrolases, since cathepsins B and D are major processing enzymes that are essential for lysosomal hydrolases in addition to proteases for the degradation of proteins. As a result, autolysosomes and autophagosomes significantly accumulated under starvation conditions in the presence of E64d and pepstatin A [10]. If PK-hLC3 reflects autolysosomes as the green-negative, but far-red-positive, fluorescent puncta, the far-red, single, fluorescent puncta of PK-hLC3 would accumulate in the presence of these inhibitors under starvation conditions. Huh7.5.1 cells expressing PK-hLC3 were incubated for 4 h under starvation conditions in the presence of these inhibitors, and the puncta of intracellular fluorescence in the cells were monitored. The green-negative, but far-red-positive, puncta of PK-hLC3 were significantly increased in the cells (Fig. 5Da–Dd). In contrast, few puncta were observed in the cells expressing PK-hLC3ΔG under the same conditions (Fig. 5Ea–Ed). The mTOR-signaling pathway negatively regulates autophagy. Rapamycin/sirolimus inhibits activities of mTORC1 complex *via* FKBP12, resulting in an induction of autophagy [30], [31]. To investigate whether fluorescent puncta of PK-hLC3 are increased by rapamycin-induced autophagy, Huh7.5.1 cells expressing PK-hLC3 were incubated for 6 h under nutrient-rich conditions in the presence of 100 nM rapamycin, and the puncta of intracellular fluorescence in the cells were monitored (Fig. 5Fa–Fd). The green-negative, but far-red-positive, fluorescent puncta of PK-hLC3 and the double-positive puncta were increased in the cells.

We further investigated the formation of puncta of PK-hLC3 using a highly potent, selective and ATP-competitive mTOR inhibitor, torin1, that also induces autophagy [32]. As was the case in the rapamycin-treated cells, the green-negative, but far-red-positive, puncta and the double-positive puncta of PK-hLC3 were observed when the cells expressing PK-hLC3 were treated with 100 nM torin1 for 6 h (Fig. 5Ga–Gd).

The lysosomotropic agents, ammonium chloride and chloroquine, inhibit acidification of the intracellular compartments, leading to a defect in the fusion of autophagosomes with lysosomes during autophagy [33]. These reagents simultaneously induce the vacuolation of intracellular compartments including autophagic vacuoles [34]. If PK-hLC3 is localized to autophagosomes and autolysosomes, treatment of the cells with ammonium chloride and chloroquine should result in an increase in the green and far-red, double-positive, fluorescent puncta/vacuoles. The cells expressing PK-hLC3 were treated with 20 mM ammonium chloride or 20 µg/ml chloroquine for 4 h under starvation conditions (Fig. 5Ha–Hd and Ia–Id, respectively). The green and far-red, double-positive, fluorescent puncta and vacuoles of PK-hLC3 were significantly increased. In contrast, only a few fluorescent-positive puncta and no vacuoles were observed in the cells expressing PK-hLC3ΔG in the presence of chloroquine (Fig. 5Ja–Jd). These results suggested that PK-hLC3 is suitable for monitoring autophagosomes and their maturation step.

## Discussion

By comparison with EGFP and mWasabi, the super-ecliptic pHluorin, a pH-sensitive mutant GFP, was more sensitive to acidic pH. The green fluorescence of pHluorin was significantly decreased at pH 6.5, and the fluorescence was further decreased to a level similar to the autofluorescence of cells at pH 5.5–6.0. The pHluorin-mKate2 tandem fluorescent protein showed green and far-red fluorescence at pH 7.2, while it showed far-red, single-positive, fluorescence at pH 5.5–6.0, indicating the pHluorin-mKate2 tandem fluorescent protein is suitable as a tandem-fluorescence protein-tag for the monitoring of autophagosome maturation. The PK-hLC3 formed an E1-substrate intermediate with Atg7, and an E2-substrate intermediate with Atg3, suggesting that fluorescent, protein-tagged LC3 is a modifier of LC3-lipidation. The PK-hLC3 formed green and far-red, fluorescent puncta, and far-red, single-positive, fluorescent puncta were observed under starvation conditions. When autolysosomes were accumulated under starvation conditions by treatment with E64d and pepstatin A, far-red, single-positive puncta were significantly increased. Under the same conditions, few puncta of PK-hLC3ΔG were observed. These results suggested that PK-hLC3 and its negative control, PK-hLC3ΔG, are suitable to monitor the mammalian autophagosomal maturation step.

In general, the pH of the cytosol is 7.4, that of the endoplasmic reticulum is 7.0, that of the *cis*-Golgi is 6.5, the pH of secretory vesicles is 5.0–6.0, early endosomal pH is 5.9–6.8, and late endosomal pH is 5.4–5.6. When autophagosomes are formed, the intra-autophagosomal pH is considered to be near that of the cytosol. During the autophagosome-lysosome fusion, the intra-autophagosomal pH turns acidic. Considering that the green fluorescence of pHluorin is significantly decreased at pH 6.5, the green fluorescent puncta of PK-hLC3 will reflect autophagosomes and an early stage of autophagosome-lysosome fusion. Therefore, when using PK-hLC3, the signals of autophagosomes will be detected as green and far-red double positive puncta in a more sensitive manner. Using PK-hLC3ΔG as a negative control, artificial fluorescent signals that are independent of autophagy will be excluded in the cells.

Autophagy in the tissues of transgenic mice uses EGFP-LC3. Our results suggested that the puncta of EGFP-LC3 in the mouse tissues tends to be overestimated as autophagosome formation. Now we are generating PK-hLC3- and PK-hLC3ΔG-transgenic mice. These mice will confer the problems derived from EGFP and EGFP-LC3 to further the study of autophagy. In future studies, we will report autophagic events in mouse tissues using these transgenic mice.

## Materials and Methods

### Cells, Media, Materials, and Antibodies

KOD-plus- (KOD-201) was employed for high-fidelity polymerase chain reactions (TOYOBO). Huh7.5.1 cells derived from the Huh7 cell line (ATCC CCL-185) were cultured in a Dulbecco's modified Eagle Medium (DMEM; Wako, 045-30285) containing 10% fetal calf serum (JRH biosciences/SIGMA, 12603C) and 1% nonessential amino acids (Invitrogen, 11140050). Polyclonal antibodies against human LC3 was described previously [10]. The mouse monoclonal antibody, clone M2, against FLAG peptide (DYKDDDDK) (F1804), ammonium chloride (254134), and chloroquine diphosphate salt (C6628) were purchased from SIGMA-ALDRICH, the mouse monoclonal antibodies against GAPDH (ab8245) were from Abcam, and the rabbit monoclonal antibody against Myc epitope tag (2278) was from Cell Signaling Technology. Protein concentrations were determined using the bicinchoninic acid protein assay reagent (Pierce, 23225). E64d (4321-v) and pepstatin A (4397-v) were purchased from Peptide Institute, and the rapamycin (#tlrl-rap) was from InvivoGen. Torin1 was a kind gift from Dr. Nathanael S. Gray at the Dana Farber Cancer Institute and Dr. David Sabatini at the Whitehead Institute for Biomedical Research. To introduce the plasmid into the cells, FuGENE HD transfection reagent (E2311) was used (Promega).

### Construction of plasmids for the expression of pHluorin-based proteins

The plasmid containing DNA fragments encoding super-ecliptic pHluorin was kindly provided by Dr. James Edward Rothman [24]. pEX-GFPhLC3wt, pEX-GFPhLC3ΔG, pTag2B-hATG7, pTag2B-hATG7C572S, and pTag3B-hATG3C264S were described previously [8], [12], [35]. A pmKate2-C plasmid containing a DNA fragment of mKate2 (FP181) was purchased from Evrogen, the pmWasabi-C plasmid (ABP-FP-WCNCS10) was from Allele Biotechnology, and pEGFP-C1 (#6084-1) was from Clontech/TAKARA. FUGW (Addgene plasmid 14883) plasmid for 3^rd^ generation lentiviral plasmid with the human polyubiquitin C (hUbC) promoter was obtained from Addgene [25]. Using two primers, pHluorin-NheI-F (5′-GCT AGC GCC ACC ATG AGT AAA GGA GAA GAA CTT TTC ACT GGA GTT G-3′) and pHluorin-GS-Bgl2-Rv (5′-AGA TCT ACC TCC TCC ACC TTT GTA TAG TTC ATC CAT GCC ATG TGT AAT C-3′), a DNA fragment was amplified via high-fidelity polymerase chain reaction using the pHluorin plasmid as a template to introduce the *Nhe*I site and the Kozak sequence just prior to the start codon of pHluorin and the Gly-Gly-Gly-Ser sequence and *Bgl*II site prior to the stop codon of pHluorin, and the amplified fragment was cloned into a pCRII-TOPO plasmid using a TA-cloning kit (K2050, Life Tech.), designated pCR-pHluorin. To introduce the *Bam*HI site before the start codon of mKate2 and the Gly-Gly-Gly-Ser linker and the *Xho*I-*Bgl*II site before the stop codon of mKate2, a DNA fragment was amplified via high-fidelity polymerase chain reaction using two primers, mKate2-BamHI-F (5′-GGA TCC ATG GTG AGC GAG CTG ATT AAG GAG AAC ATG CAC-3′) and mKate2GS-BglII-Rv (5′-CTC GAG ATC TGA GTC CGG AAC CTC CTC CAC CTC TGT G-3′) with pmKate2-C. The amplified DNA fragment was introduced into a pCRII-TOPO plasmid, designated pCR-mKate2. The *Nhe*I-*Bgl*II DNA fragment containing the open reading frame of EGFP in the pEGFP-C1 was replaced with the *Nhe*I-*Bgl*II DNA fragment containing the open reading frame of pHluorin in the pCR-pHluorin for the expression of pHluorin under the control of a cytomegalovirus immediate early promoter, designated pHluorin-G. The *Bam*HI-*Bgl*II DNA fragment containing mKate2 of pCR-mKate2 was inserted into the *Bgl*II site of the pHluorin-G plasmid for the expression of a pHluorin-mKate2 fusion protein under the control of a cytomegalovirus immediate early promoter, designated pHmK-G. For the expression of wild type PK-hLC3, the *Nhe*I-*Bgl*II DNA fragment containing the open reading frame of EGFP of pEX-GFP-hLC3WT (Addgene plasmid 24987, Addgene) [12] was replaced with the *Nhe*I-*Bgl*II DNA fragment containing the open reading frame of pHluorin-mKate2 fusion protein derived from pHmK-G plasmid, and the resultant plasmid was designated pEX-PK-hLC3. For the expression of mutant PK-hLC3ΔG, the *Nhe*I-*Bgl*II EGFP DNA fragment of pEX-GFP-hLC3ΔG (Addgene plasmid 24988, Addgene) [12] was replaced with the *Nhe*I-*Bgl*II DNA fragment encoding pHluorin-mKate2 fusion protein, and the resultant plasmid was designated pEX-PK-hLC3ΔG. For the lentiviral packaging system for the expression of PK-hLC3 fusion protein, the DNA fragment was amplified via a high-fidelity polymerase chain reaction using pEX-PK-hLC3, pH-Bam-Nhe-F (5′-AAA GGA TCC GCT AGC GCC ACC ATG AGT AAA GGA GAA G-3′), and hLC3-RI-Rv (5′-AAA GAA TTC TTA CAC TGA CAA TTT CAT CCC GAA CG-3′) primers. After the digestion of the amplified DNA fragment with *Bam*HI-*Eco*RI, the fragment was inserted into the *Bam*HI-*Eco*RI site of FUGW. The resultant plasmid was designated FUGW-PK-hLC3. For a lentiviral packaging system for the expression of mutant PK-hLC3ΔG, the hLC3ΔG-RI-Rv (5′-AAA GAA TTC TTA CAC TGA CAA TTT CAT GAA CG-3′) primer was employed instead of hLC3-RI-Rv, and the resultant plasmid was designated FUGW-PK-hLC3ΔG.

### Immunoblotting analyses

Cells were washed twice in phosphate-buffered saline, lysed in lysis buffer (10 mM sodium phosphate, pH 7.2, 150 mM NaCl, and 1% sodium dodecyl sulfate) containing a Complete protease-inhibitor cocktail (Roche Diagnostics, 1697498). Proteins (10 µg) of the lysate were separated on sodium dodecyl sulfate polyacrylamide gel electrophoresis (SDS-PAGE). After transferring the proteins to a polyvinylidine difluoride membrane using a Trans-Blot SD transfer cell (Bio-Rad, 170-3940), FLAG-Atg7, Myc-Atg3, LC3 and GAPDH in the lysate were recognized using the appropriate antibodies. A chemiluminescent method was carried out according to standard protocols with SuperSignal West Dura Extended Duration Substrate (Pierce, 34075) or SuperSignal West Pico Chemiluminescent Substrate (Pierce, 34077).

### Fluorescent Microscopy

Cells were fixed in a fixation solution (phosphate buffered saline containing 4% paraformaldehyde) at room temperature for 5 min, and permeabilized in a phosphate-buffered saline containing 50 µg/ml digitonin. After cells were buffered in 20 mM citrate phosphate buffer at pH 5.5, 6.0, and 6.5 containing 150 mM NaCl when indicated, the fluorescence of the fluorescent proteins was monitored using a Biozero BZ-8000 microscope (KEYENCE, Tokyo, Japan).

### Laser scanning confocal microscopy

The fluorescence of the fluorescent proteins in the cells expressing PK-hLC3 was monitored using a Olympus FluoView FV1000 confocal laser scanning microscope. Pearson's correlation coefficient (PCC) analysis with Costes' method [36] was employed to estimate the colocalization of mKate2 signals with pHluorin signals of at least 20 independent images using ImageJ software (http://imagej.nih.gov/ij/) [37] with a JACoP (Just Another Colocalisation) plugin (http://www.blackwell-synergy.com/doi/pdf/10.1111/j.1365-2818.2006.01706.x) [38].
